# Knowledge, practices and perceptions of geo-helminthes infection among parents of pre-school age children of coastal region, Kenya

**DOI:** 10.1371/journal.pntd.0005514

**Published:** 2017-03-30

**Authors:** Janet Masaku, Faith Mwende, Gladys Odhiambo, Rosemary Musuva, Elizabeth Matey, Jimmy H. Kihara, Isaac G. Thuita, Doris W. Njomo

**Affiliations:** 1 Eastern & Southern Africa Centre of International Parasite Control (ESACIPAC), Kenya Medical Research Institute (KEMRI), Nairobi, Kenya; 2 Centre for Global Health Research (CGHR) Kenya Medical Research Institute (KEMRI), Kisumu, Kenya; 3 Centre for Microbiology Research (CMR), Kenya Medical Research Institute (KEMRI), Nairobi, Kenya; 4 Directorate of Basic Education, Early Childhood Education Section, Ministry of Education, Nairobi, Kenya; 5 Division of Vector Borne Diseases, Ministry of Health, Nairobi, Kenya; University of Melbourne, AUSTRALIA

## Abstract

**Background:**

Soil-transmitted helminthes (STHs) are common human parasitic diseases in most of the developing world particularly in Kenya. The ongoing National School-Based Deworming Programme (NSBDP) was launched in 2012 and is currently targeting 28 of the 47 endemic Counties. In an effort to improve treatment intervention strategies among Pre-School Age Children (PSAC) attending Early Childhood Development Centres (ECDC), we sought to assess parents’ knowledge, perceptions and practices on worm infection.

**Methodology:**

We conducted a qualitative cross-sectional study in four endemic sub-counties of two counties of coastal region of Kenya. A total of 20 focus group discussions (FGDs) categorized by gender were conducted among parents of pre-school age children. Participants were purposively selected based on homogenous characteristics with the saturation model determining the number of focus group discussions conducted. The data collected was analyzed manually by study themes.

**Findings:**

**The** majority of the parents had knowledge on worms and modes of transmission of the parasitic infections among the pre-school children. Also, most of the participants knew the causes of worm infection and the pre- disposing factors mentioned included poor hygiene and sanitation practices. Due to poor knowledge of signs and symptoms, misconceptions about the drugs administered during the NSBDP were common with a large majority of the parents indicating that the drugs were ineffective in worm control. The findings also indicated that most of the participants sought medical care on the onset of the signs and symptoms of worm infestation and preferred services provided at public health facilities as opposed to private health facilities or buying drugs from the local market citing mistrust of such services. Cultural beliefs, high cost of building and availability of vast pieces of land for human waste disposal were factors that contributed to low or lack of latrine ownership and usage by a large majority of the respondents.

**Conclusions:**

Our results show that to a large extent the parents of the pre-school age children have information on worm infections. However, some cultural beliefs and practices on the pathology and mode of transmission mentioned could be a hindrance to prevention and control efforts. There is need to implement health promotion campaigns to strengthen the impact of control strategies and reduce infection.

## Author summary

Soil-transmitted helminthes commonly known as worms, form part of the Neglected tropical diseases which are associated with substantial acute and chronic morbidity, particularly among children. Hookworm occurs throughout most of the country, with areas of highest prevalence being found in South-Western Kenya and the Coast Region. Regular administration of anthelmintic drugs has been strongly supported as an effective control strategy. Soil-transmitted helminthes can be controlled using three key approaches which include improved sanitation, mass treatment with albendazole and health education. Additional interventions for infection prevention include, access to safe water, environmental management and promotion of hygiene. However, the success of control initiatives involving the community depends on the level of the communities uptake of the programme, which is linked to the understanding of the community knowledge, practices and perceptions towards the disease. Therefore this study identified the gaps that should be addressed to contribute towards strengthening the parasitic infections control interventions in the area. The findings revealed high knowledge on worms and limited knowledge on signs and symptoms on the parasitic infections. These findings are valuable in designing approaches towards enhancing health outcomes in the community like implementing a community treatment and prevention programme to ensure effective control measures.

## Background

Neglected tropical diseases (NTDs) are a cluster of tropical diseases that affect more than one billion people worldwide, mainly among poor populations. Soil-transmitted helminthes (STHs) which form part of the NTDs are associated with substantial acute and chronic morbidity, particularly among children, in whom the highest intensities of infection are found [1]. According to the 2010 Global Burden of Disease study, the STHs spp *Ascaris lumbricoides*, *Trichuris trichiura* and hookworm, contribute the greatest disease burden among the NTDs, causing an estimated 4.98 million years lived with disability each year [2–3]. Chronic infections can have insidious effects on childhood development, including growth and cognitive development, whilst heavy infections may result in serious clinical disease. Both chronic and intense infections are most common in school-age children who are the natural targets for school-based chemotherapy programmes.

In Kenya, the prevalence of STHs is prominently attributed to *Ascaris lumbricoides*, hookworm and *Trichuris trichiura* [4]. It is estimated that approximately 10 million Kenyans are infected with STHs and over 12 million people living in rural endemic areas in the country are at risk of infection with these parasites [5]. Hookworm occurs throughout most of the country, with areas of highest prevalence being found in south-western Kenya and in Kilifi and Kwale Counties in the Coastal Region [4]. *Ascaris lumbricoides* and *Trichuris trichiura* are most prevalent in western Kenya and in coastal areas, especially Lamu and Tana River Counties [6].

Regular mass administration of anthelmintic drugs which usually takes place outside the health care settings has been strongly supported by the World Health Organization (WHO) as an effective control strategy to affected populations especially on school going children. Millions of school-aged children have received anthelmintic treatment (albendazole or mebendazole against soil-transmitted helminthiasis) to good effect [7–9]. However, lack of sanitation, poor water supply and unclean practices contribute to the spread of all intestinal helminthes infections in the community. In recognition of the burden helminthes infections impose on human populations, particularly the poor, major intervention programmes have been launched to control them [10]. Although reductions in the prevalence and morbidity have been achieved through chemotherapy, it is generally accepted that improvements in domestic water supplies, environmental sanitation, health education, access to health services for diagnosis and treatment must be integrated in control and elimination programmes to assure their effectiveness [11].

In the short term, it is questionable whether increased availability of latrines can be expected to drastically decrease the prevalence of helminthes infection. Until an overall improvement of sanitation can be achieved, the use of community treatment by chemotherapy and improvement of personal hygiene by health education are advocated for as effective and affordable measures for controlling helminthes infections. Safe, inexpensive and effective anthelminthic are now widely available, [12–13] but the success of a chemotherapy programme will depend upon the selection of the target group and type of anthelminthic [14].

Health education is aimed at reducing transmission and re-infection by encouraging health behaviours. The main purpose is to reduce contamination of soil, promoting the use of latrine and hygienic behaviors [15].Periodic deworming certainly can attain a stable reduction in transmission if deworming continues indefinitely. Health education can be provided simply and economically, and its benefit goes beyond control of helminthes infections [5]. For health education activities to be effective they will also need the identification of the target audience and the formulation of clear messages, which take into account local perceptions and attitudes to bring about behavior change [16]. Furthermore, the role played by the target population is of great importance. If the members of the community are aware of the negative effects of intestinal helminthes on the health of their children, they will be more likely to support and sustain uptake of MDA intervention measures. It is more or less of a challenge in the coastal region of Kenya and there is evidence that more education and knowledge to the community members would improve the uptake [17]. Nevertheless, there are risk factors which community members are able to control without much effort, like washing hands before eating, drinking boiled or treated water, wearing shoes and eating well cooked food. Recent studies support that both individual and community perceptions and attitudes of parasitic worm infections and their prevention and treatment are important factors [16, 18–19]. Although it is recognized that the control and prevention of parasitic diseases depend upon adequate knowledge of human behaviour [18] the literature on this subject is limited and few studies have considered people's perceptions and attitudes toward worm infection, treatment and control [20–21].

In Kenya, the National School-Based Deworming Programme (NSBDP) was launched in 2012 and is currently targeting 28 of the 47 endemic Counties. To date school-age children have received at least 3 annual rounds of treatment with albendazole (2–14 years) using trained school teachers. However, the Programme does not consider the pre-school teachers as eligible for training to conduct deworming of the children. This is mainly because the pre-school teachers are not employed by the government and they cannot take responsibility for any adverse outcome of the treatment. The Programme therefore requires that pre-school children whose centres are not within (standalone) the primary schools be taken to the nearby primary schools to receive treatment from the trained teachers. This may have negative implications in terms of accessing the nearest school due to the distance covered and majority of the pre-school children are likely to miss the treatment. Results of the baseline stool survey conducted in 50 pre-schools of 5 sub-counties of the Coastal Region in the year 2012 by Kenya Medical Research Institute (KEMRI), prior to deworming activity show that the prevalence of worm infestation requires annual mass treatment. Currently the prevalence of STH in some pre-schools in Kwale sub-county is 27.8%, in Malindi sub-county, 44.5% and in Msambweni sub-county, 66.7% [22]. This study assessed parents of pre-school age children’s awareness of existence, signs and symptoms, causes, transmission, control and risk factors of STHs as well perceptions, health seeking behavior and practices that affect STHs control. The study identified gaps that should be addressed to contribute towards strengthening STHs control interventions in the area.

## Materials and methods

### Ethics statement

Ethical clearance was received from the Kenya Medical Research Institute (KEMRI)/National Ethical Review Board (Protocol number 2547). Permission was obtained from the County Directors of Health of Kwale and Kilifi Counties. Permission was also sought from the area assistant chief and village elders who were notified about the study. A written informed consent was sought from all the study participants. All the participants were adults above the age of 18 years and therefore no parents/guardians were expected to give consent on behalf of a minor. All information given by the study participants was kept confidential and anonymity was highly observed. No personal identifiers were used during data entry, analysis and presentation.

### Study site and healthcare delivery

The study was conducted in four endemic sub-counties (Matuga, Msambweni, Lunga Lunga and Malindi) in coastal Kenya. Three of the sub-counties are located in Kwale County while Malindi sub-county is located in Kilifi County. All the sub-counties are located along the coastal region of Kenya, and had been participating in the NSBDP. They were actually not a representative of other Counties involved in the NSBDP, but were purposively selected due to STHs endemicity and the long distance covered from homesteads to the nearest school due to marginalization [22]. Apart from the NSBDP, there was an ongoing community MDA (targeting adults and school going children) in Kwale County only during the time of the study. However, this did not affect the outcome of this study because the community MDA programme was in its initial stages of implementation.

Kwale County is located, 40 kilometers (kms) south of Mombasa, the second largest city in Kenya, which has an area of 8360 km2 with a population of 649,931 persons [23]. In terms of climate, the county is hot and dry from January to March and relatively cool from June to August. The rainfall pattern is bimodal with long rains normally occurring between mid-March and June while the short rains occur between October and December. Subsistence farming of food crops, including maize, cowpeas and cassava is done mainly for domestic consumption. Tourism is also done in the urban setting of the county and fishing is mainly done by those living along the Indian Ocean.

Malindi Sub County is located 120 kilometers north east of Mombasa. Tourism and fishing are major economic activities due to its proximity to the Indian Ocean. However, it is rated among one of the poorest sub-counties in Kenya due to poor rainfall [23]. It covers a geographical area of 7,605 km2 with a total population of 384,643 [23].

Kwale County has 3 hospitals, 5 health centers, 37 government dispensaries and 3 private dispensaries. Accessibility of health services is however low. Majority of the population live over 5 kms away from the nearest health facility. Shortage of drugs and lack of diagnostic facilities adversely affect provision of quality health care. Cost of health care system is also a barrier of access to services. The doctor/patient ratio stands at 1:82,690 which in itself is telling of services offered due to shortage of staff in the health facilities. The utilization of health facility for child delivery stands at 32% [24], the main reasons for low levels of use being distances to the nearest health facilities and low socio-economic status. Malindi sub-county has 3 hospitals (1government and 2 private); 24 dispensaries (17 governments and 7 NGO) and 4 private chemists. The average distance to the nearest health facility for urban areas is 1 km and 3 kms for rural areas. Most of the health facilities are therefore not accessible to the majority of the population. High poverty levels, cost sharing and long distances inhibit people from visiting these facilities. The doctor/patient ratio is 1:19,502. The most prevalent diseases are malaria, respiratory diseases, diarrhea, intestinal worms, STIs, anemia and eye infections.

### Study design and setting

This was a qualitative cross-sectional study. A single discussion was held with each focus group, between the dates of 13^th^ October and 3^rd^ November 2015 in the four endemic sub counties. Twenty FGDs were conducted with parents of pre-school age children to gauge their perceptions, knowledge and practices of STHs infection and control. In the three sub-counties of Kwale County, four villages were randomly selected from each sub-county totaling to twelve. While, in Malindi sub-county located in Kilifi County, eight villages were also randomly selected. This was to ensure inclusiveness and representation in the Malindi sub-county which had more villages compared to other sub-counties. In each selected village, one FGD discussion was conducted for the assigned gender (male or female FGD per village separately). Hence ten male FGDs and ten female FGDs were conducted in all the four sub-counties. The reason for separation of the FGDs by sex was due to the rural setting of the study area, whereby gender roles are clearly defined and most female study participants were not able to express themselves freely in the presence of their male counterparts. The participants were purposively selected based on their age, level of education, gender, having a pre-school going child and living in the targeted villages. Saturation model was used to determine the number of focus group discussions conducted [25]. Data was collected in *Swahili*, the local language. Further screening was done on site to make sure that participants met the inclusion criteria before obtaining informed consent, and that they were fully representative of the different villages. The research team came up with a semi-structured FGD guide that provided a general overview of the topics. Discussions were steered by a moderator who was part of the field team and had undergone training before the data collection exercise. However, the direction the discussion took depended on the participant’s responses and areas that the moderator felt needed probing. The FGD guide was first pilot tested in an area with similar social-economic characteristics to the study sites. The discussion guide covered: common diseases experienced in the study sites, knowledge of signs and symptoms, causes, treatment, and prevention of intestinal helminths. Water use and sanitation practices and health seeking behavior were also discussed. FGDs sessions were tape-recorded and transcribed. The information was used to create a detailed reconstruction of respondents’ knowledge, practices and perceptions with regards to STHs infections.

### Study population

The FGDs participants included single sex adult (18 years and above) male and female respondents who shared similar characteristics, like gender, age, knowledge and cultural practices. Each FGD contained a minimum of 8 and a maximum of 12 participants and standard procedures were adhered to [26]. Twenty focus group discussions were conducted with adult community members (male and female separately). The inclusion criteria for the parents were to have children attending pre-school, to be living in the targeted villages, knowledgeable and willingness to give consent to participate in the study. Community health extension workers (CHEWs), who are not employed by the government but have basic knowledge and usually participate voluntary on health activities in their respective villages were trained and recruited by the study to help in community mobilization. Data was voice recorded and transcribed, coded and thematically analyzed manually based on study themes.

### Data management and analysis

All the qualitative data was transcribed verbatim and the text typed into the computer spreadsheet. The data cleaning and analysis was done manually. A code sheet was created following the focus group discussions after which, the textual data was coded into selected themes and a master sheet analysis was carried out, giving all the responses from the FGDs a theme. Thematic analysis was used where responses were categorized into themes and then ideas formulated by looking at the patterns of responses [27]. Analyzed data was presented in text form. Quantitative data from the socio-demographic profiles was analyzed using excel spreadsheets.

## Results

The overall characteristics of the study participants were (N = 203); 49.3% were females. The majority age range of the study participants was 30–34 years 19.2%. Not all the FGDs participants were educated with 51.7% having primary education. 52.7% of the FGDs participants were of Muslim religion while Christians were 43.8%. Farming was the dominant occupation by most of the FGDs participant with 54.7%. (Table 1).

**Table 1:** Socio-demographic characteristics of the study participants in the FGDs.

Description	Frequency (*N* = 203)	Percentage (%)
Gender		
Male	89	43.8
Female	100	49.3
Missing	14	6.9
Age in years		
15–19	2	1.0
20–24	36	17.7
25–29	30	14.8
30–34	39	19.2
35–39	35	17.2
40–44	18	8.9
45–49	18	8.9
≥ 50	24	11.8
Missing	1	0.5
Educational level		
Primary education*	105	51.7
Secondary education*	14	6.9
None	47	23.2
Missing	37	18.2
Religion		
Christianity	89	43.8
Islam	107	52.7
None	4	2.0
Missing	3	1.5
Occupation		
Business	40	19.7
Farming	111	54.7
Fishing/Fish monger	3	1.5
Housewife	27	13.3
Casual laborer	9	4.4
Religious leader (Pastor or Imam)	3	1.5
Community health volunteer	1	0.5
Skilled laborer	2	1.0
Village elder	1	0.5
Teacher	3	1.5
Missing	3	1.5

* Includes people who received some education but may not have completed this level

### Knowledge and level of awareness on STHs infection

#### Common diseases, local name and where they heard about STHs

STHs were among the list of the most common illnesses experienced by pre-school age children according to a majority of the parents in the FGDs. Other common diseases mentioned were skin diseases and ringworms. Various local names for STHs were given as: *Minyoo*, *Mishang’o*, *and Minyolo* for hookworm and round worms. Participants in the focus discussions declared having heard about STHs before, especially during schooling. Generally the level of awareness was high with half (n = 10) of the FGDs participants indicating that they could identify the various types of worms like hookworms, round worms and tapeworms. A minority of the participants in a tenth (n = 2) of the FGDs however, indicated that they had never heard about STHs at all. A 30 years old female participant stated that:

*“It is a long time ago, I was still in school. I was very young and when I went to the toilet I saw something coming out and I thought it was a snake so my father told me they were worms”*.

### Awareness on causes of transmission

A majority of the study participants had inadequate knowledge on modes of worm transmission and reported that worms were caused by eating cold food and bathing in dams. Also, most of the participants reported that the disease was caused by walking bare footed, drinking untreated water, eating soil, open defecation, eating undercooked vegetables and meat, meaning they knew the source of infection or mode of transmission of STHs. A 60 years old male participant stated that:

*“We get worms from the food and also from the water we drink. The water we drink here isn’t from taps, it is from ponds. We mainly use rain water. We wait for rain to pour down, and then we go to dig up somewhere. That water has a lot of dirt. It has typhoid, it has worms. I mean it just has many things that contribute”*.

### Signs and symptoms of STHs, knowledge gap and associated misconceptions

Study participants in four-fifths (n = 16) of the FGDs had knowledge on worms and limited knowledge on signs and symptoms caused by the parasitic infections among the pre-school age children. Weight loss, pall or anal itching, abdominal pain, diarrhea, enlarged stomach, ring worms, coughing, vomiting, fatigue, lack of appetite and craving for soil were listed as major signs of worm infection. A 49 years old male FGD participant stated that:

*“Sometimes when you give a child food they take a small portion then they leave, after a while they come back that they are hungry. Also the itchiness that they experience in their anus makes you know they have worms”*.

A large number of the study participants in three quarters (n = 15) of the FGDs felt that they were poorly informed about worms and they were susceptible to worm infection. The participants further indicated that they would be interested in learning more about STHs in such areas as signs and symptoms, causes, cure, drugs, how it is spread, risk susceptibility, prevention and control. A female FGD participant aged 49 years stated that:

*“I would like to know how one gets infected by the worms, the causes of these worms, the signs and symptoms, so that I may tell when my child is infected by worms”*.

Lack of adequate knowledge on worm infection was exemplified by the reported signs and symptoms and intervention measures that were not linked to infection. A large number of the study participants perceived that ring worms which are fungal infections were signs and symptoms of STHs infection leading to the misconceptions that albendazole which is the common drug administered to pre-school age children during mass drug administration (MDAs) was ineffective. A 30 years old farmer female participant stated that:

*We have seen that they don’t work because the disease is still there. The ringworms don’t get healed. If these ringworms could go away then we can say the drugs are effective but kids are getting the drugs but nothing is changing”*.

Other misconceptions on causes and signs and symptoms of worms infection mentioned by the study participants were, sharing of food among children, eating cold and left-over food, having skin rashes or diseases, sharing combs and hand washing basins, bathing and drinking un-boiled water. A 37 year old female participant stated that:

*“The small worms which are white in color we call them ringworm, when little children sleep they come through the anal. Also the ringworms are many, in fact I think they cause the hair to come out. It is not only on the head but the body, you see round thing on the arms and if they scratch it becomes a wound”*.

One of the male FGD participant further indicated that worms can cause syphilis. Also, a minority of the study participants in one-tenth (n = 2) of the FGDs felt that worms can be caused by having many sexual partners probably due to urinating blood which is caused by *Schistosoma haematobium*: A 52 year old male participant stated that;

*“All this comes as a result of our sinful nature because from here, I go and get intimate with a woman, who is someone else’s wife and maybe that woman has this disease. So you know I have contracted that disease. And I go and infect my wife”*.

### Awareness about control and prevention measures

With regard to ways of preventing infection with STHs a large number of the study participants in all the FGDs indicated that washing hands, wearing protective clothing while farming and drinking boiled or treated water were measures of infection control. Other measures given were proper human waste disposal, treatment with drugs and general personal hygiene. Participants also mentioned that wearing shoes, health education, building and using toilets/latrines would help in combating the infection. A 34 year old female FGD participant stated that:

*“I think personal hygiene is important to prevent the worms which entails, clean drinking water, washing vegetables and fruits before use, maintaining cleanliness around our homes and making sure we wear shoes before going to the latrine”*.

### Perceptions of community members on worm’s infection

Participants in two-fifths (n = 8) of the FGDs perceived worms to be ubiquitous, with majority considering that all people were likely to become infected. A half (n = 10) of the FGDs participants reported that they knew someone who could be having worms infestation. This was however based on the knowledge of the misconceptions of the signs and symptoms. When asked about the most affected age group, a large number of the participants in all the FGDs indicated that children were more often infected compared to all other age groups due to their poor hygiene habits, eating soil and walking barefooted. A 60 years old male participant stated that:

*“These ones with very low age like two years or three years especially these in nursery school, I think are the ones who get worms. This age likes to get into such kind of environment like eating soil and walking bare footed. That’s why”*.

Regardless of age, gender and location the participants in this study had almost similar level of knowledge, attitude and practices on STHs infection. However, when asked about which gender was more at risk of being infected with worms, most of the FGDs participants perceived that women were more at risk of getting worms compared to male due to practices like eating soil during pregnancy, farming, taking care of the children and their general unhygienic practices. A 53 year old male FGD participant stated that:

*“I think its women, they like eating soil when expectant. Another thing is she will take her baby and wash her. The dirt is still on her hands. She takes a bowl, puts in some vegetables. She washes her hands, but not properly. So her nails still have the dirt”*.

A large number of the participants in all the FGDs considered farmers as the most affected occupational group due to much contact with soil. Participants in nine tenth (n = 18) of the FGDs perceived worms as a serious health problem, particularly among children, although the infection was considered very common and no social stigma was associated with the infestation condition. A 34 years old male participant stated that:

*“You know here worms are very common it is like our religion. The one with worms is not considered like there is anything wrong. It is just normal. People don’t suspect him of anything or exclude him. It’s just a normal thing”*.

With regard to worm infection, participants in one quarter (n = 5) of the FGDs felt that they could be infected with worms. When asked about how they would react if they had worms, only two fifth (n = 8) of the participants in the FGDs mentioned that they would accept their condition and seek medical attention. However, a minority of the participants in a quarter (n = 5) of the FGDs reported that they would feel bad and ashamed wondering where they would have been infected from. A 37 years old female participant stated that:

*“I will feel bad because the doctor has said I have worms and I am an adult so where could I have been infected by the worms?”*.

When asked what would really worry them if they suspected that they had worms, participants in more than half (n = 11) of the FGDs reported their worry would be that worms can suck blood, bring ill health, body weakness and discomfort. Nevertheless, a minority in one fifth (n = 4) of the participants reported that their worry would be that worms can lead to death. A 42 years old housewife female participant stated that:

*“What will worry me is that the stomach pains not knowing what it is. I think the worms eat things in the stomach so when they are done I will surely die. So I will be worried and go to the doctor”*.

Participants in one half (n = 10) of the FGDs mentioned that they would confide with their spouses before seeking medical attention in case they suspected that they are infected with worms. However, a minority of the participants in one quarter (n = 5) of the FGDs reported that they would confide in the doctor first before informing anyone else. A 25 year old Male FGD participant stated that:

*“The first person will be my wife because we live in the same house, before going to the hospital she has to know where exactly am going and what it is am going to do there, after that the second person will be the doctor”*.

### Health seeking behavior on STHs

Majority of the participants in four fifths (n = 16) of the FGDs reported to prefer seeking treatment for themselves and their children in government/ public health facilities as opposed to private facilities or buying drugs from the local market. When asked the reason behind the preference of seeking treatment in government health facilities, as opposed to the private health facilities or chemists. Participants cited to have much trust in clinicians in the public health facilities compared to private health facilities or chemists whereby the drugs administered may be incorrect or inactive. A male FGD aged 38 years reported that:

*“Going to the government hospital is better because you get tested but in the chemist they can give you any drug because they do not test you and they are there for business so the government hospital is the better choice”*.

Nevertheless, due to lack of finances, long queues and long distances to the public health facility, half (n = 10) of the FGDs study participants reported to be buying drugs from the local chemist or pharmacy to avert those problems and cut down on the cost of treatment. A 34 years old farmer male participant stated that:

*“If I think I have worms, when I think about the trip to the government hospital to see the doctor. Then he gives me the container to put my stool in it, it is a process. I would rather go to the chemist directly. I will ask the person in the chemist to give me deworming drugs”*.

With regard to acceptability of worm treatment, participants in four fifths (n = 16) of the FGDs reported that the treatment was acceptable to the community members and the infection could be controlled with proper medication. Notwithstanding, distance to the nearest health facilities due to poor road network and lack of transport, lack of drugs, poor equipped laboratories in the local health facilities and lack of interest by health personnel to carry out stool tests were limiting factors for seeking treatment in three fifths (n = 12) of the FGDs participants as reported below. A male FGD participant aged 39 years old stated that:

*“Sometimes you go to the hospital and you are told to go and buy drugs from the chemist. There are no drugs. So you wonder what kind of a hospital it is. You have already used money for transport, yet there are no drugs”*.

In addition, a minority of the participants in one quarter (n = 5) of the FGDs reported that they would result to other modes of treatment like taking herbal medication, praying and visiting a herbalist in case they did not get well or did not have the finances. Two of the herbal trees mentioned by the participants were *Mwarubaini* and *pepper* which could heal worms as stated below by a 40 year old female FGD participant:

*“Some go to the hospital, others go to the herbalist. You can go to the hospital and see the doctor after doing his investigations he does not see any problem. But when you get home you make the herbal concoction and you get well”*.

The average cost of worm treatment was reported to be between 50 to700 Kenya shillings by more than half (n = 13) of the FGDs participants. Participants in one half (n = 10) of the FGDS felt that the cost of treatment was quite high and a deterrent to accessing worm treatment considering their low levels of income in their local rural setting. A 38 years old female farmer stated that:

*“Treating worms is very costly because there is a long process to it. First your stool has to be checked and you pay for it, then they give you drugs which are even more expensive. The drugs they give you are not even effective they tell you to go back after 3 months but you do not even go back”*.

In regard to time of seeking medication, participants in three-quarters (n = 15) of the FGDs reported that they would seek medication at the onset of the signs and symptoms of worm infestation indicating that delaying would result to serious disease progression which could harm their bodies, and would translate to high cost of treatment and transport. A 39 year old housewife female participant stated that:

*“When you wait until the situation is critical, you will not even have the energy to walk to the hospital. So you would rather go when you have the slightest symptoms”*.

### Practices in latrine use, ownership and personal hygiene

With regard to latrine ownership and utilization, only one fifth (n = 4) of the FGDs participants admitted to own and utilize pit latrines despite having knowledge that open defecation was closely linked to worm infection by less than half (n = 9) of the FGDs participants. A male FGD participant aged 42 years stated that:

*“We get worms because most us we do not have toilets at homes, now if we just defecate anywhere a housefly can just come there touch the dirt then it infects someone and even when we step by foot we also get the worms”*.

Participants in three-quarters (n = 15) of the FGDs reported that household latrines coverage and utilization was very low due to high cost of building materials, laziness, poor soil quality, low sensitization, cultural beliefs and practices and availability of vast piece of land that could be used for human waste disposal. Some of the cultural beliefs and practices mentioned were that a father in-law cannot share a pit latrine with a daughter in-law since it is a taboo and lack of respect among the local communities (Mijikendas). Another practice was that some participants indicated that they have never used a pit latrine since their childhood and did not see the reason for owning one and utilizing it since their parents were also practicing open defecation.

A 60 year old Male FGD participant stated that:

*“There are people who say they can’t construct a toilet because there is a lot of land available for relieving oneself. Others say they can’t defecate in the same place with their daughter in law. There are many who say that. That’s in the mijikenda culture in general. People are also lazy to build toilets. Even here in town center you will not find a toilet”*.

Study participants in four fifths (n = 16) of the FGDs reported that good hygiene practices like hand washing before eating and proper human waste disposal would reduce worm infection in PSAC and the community members. Other unhygienic practices likely to increase worm infection were eating unwashed fruits, walking barefooted and drinking untreated water. A 32 year old female FGD stated that:

*“Being clean, washing utensils till they are clean, wear shoes as you visit the toilet and washing hands with soap after visiting the toilet that will help a lot”*.

None the less, there was high indication that most of the unhygienic practices mentioned were attributed to poverty, high illiteracy levels and cultural practices. A male FGD aged 38 years old stated that:

*“In our culture when you visit someone’s house then that person washes their hands first in the basin, then all of you wash in the same basin. This makes the worm disease to spread because we do not know where each one of us has come from. This practice is very unhygienic and we need to stop it, because it is the only way to stop the spread of worms”*.

## Discussion

### Knowledge on causes, symptoms and transmission of STHs infection

The present study demonstrated that a reasonable proportion of the community members had adequate level of knowledge of STHs aetiology, signs and symptoms, treatment and preventive methods, results which are similar to those of other study areas [28–29]. The results also showed that most of the parents had heard of STHs infection in their childhood, especially during schooling. Similarly, a report of a study conducted in Brazil indicated that schools were a main source of information about schistosomiasis infections [30]. With regard to the transmission of parasitic worms, a common belief according to the current study results was that the spread was primarily caused by eating soil, walking bare footed, open defecation, eating raw/cold food and raw vegetables. Acka and colleagues in a similar study however, revealed different observations that soil was an unknown source of transmission for intestinal worm infections to the community members [31].

### Misconceptions on STHs infection

Furthermore, the current study results showed that there were reported misconceptions on ring worms being part of STHs infections and the drugs given in NSBDP were ineffective. These results are similar to that of another study which reported that misconceptions were due to inadequate information on intestinal schistosomiasis infection among adults in western Kenya [32]. Moreover, the current study has also shown that there were misperceptions about worm infection and some practices such as eating cold left-over foods and bathing in untreated water. This concurs with a similar study which found that lack of adequate information on worm infection was exemplified by the suggested intervention measures that were not linked to worm infection [33]. In addition results of the current study further demonstrated that there were other misperceptions that STHs infection was associated with having many sexual partners. This could be due to the confusion and mix-up on causes and symptoms between worm infection and urinary schistosomiasis which both are endemic in the study area. Noting that urinary schistosomiasis causes blood in urine. Accordingly, these misconceptions and misperceptions call for the need of health education about the causes and prevention of STHs among these communities.

### Those perceived to be at risk of STHs infection and seriousness of the disease

According to the results of the current study, two fifths (n = 8) of the FGDs perceived worms to be everywhere, with majority considering that all people were likely to become infected. However, the study participants pointed out that farmers were the most affected occupational group due to their frequent contact with soil. In respect to gender, our findings revealed a difference in perceptions towards STHs infection between the male and female sex by the FGDs participants. Most of the community members perceived that women were more at risk of getting worms compared to male due to practices like eating soil during pregnancy, farming, taking care of the children and their general unhygienic practices. Previous findings reported that women preferred to wash clothes in rivers because of suitable washing stones were available and there was time for social interaction while doing community chores which could expose them to infection with schistomiasis [31]. Also majority of the FGDs study participants perceived worm infection to be a serious infection as they suck blood, cause general body weakness and deprive the host some nutrients and in severe cases cause death. This concurs with a similar study conducted in western Kenya, which found out that community members perceived intestinal schistosomiasis infection to be a severe infection which would lead to death [34].

### Treatment seeking behavior

Stigma is known to increase feelings of fear, shame and reduces people’s capabilities to successfully obtain appropriate treatment [35]. The results of the current study also showed that a minority of the FGDs respondents if infected with STHs, they would feel bad, a bit ashamed and surprised wondering the source infection. Nevertheless, the results of the current study, outlined that this would not deter them from seeking treatment. In this regard, community perceptions of diseases are particularly important in ensuring effective control strategy’s, as perception would affect STHs prevention and control [36]. In fact, recent evidence suggests that in the north-western part of Uganda communities are increasingly resisting to regularly take anthelmintic drugs as part of preventive chemotherapy programs targeting multiple neglected tropical diseases simultaneously [37].

The study results also showed that majority of the community members would prefer seeking treatment in a government health facility as opposed to traditional medicine or drugs sold in the local market (chemists) citing lack of trust on these options. This is inconsistent with findings elsewhere, which reported that traditional medications were frequently used, since modern treatments were either unavailable due to high cost or lack of supply [38]. Current study results also showed that most of the participants would seek medical care as soon as they felt the onset of the signs and symptoms of worm infection to avoid incurring more expenses in transport and serious complication later. Nonetheless, according to the current study results, cost of treatment was reported to be quite high which could deter them from seeking treatment for themselves or their family members when infected by worms. Other restraining factors mentioned were cost of transport to the health facilities, lack of drugs supply in health centers and long queues in the facilities. Indeed, a study conducted in Ghana reported that, people who reported not to have visited a hospital or health center for their symptoms were due to lack of money and symptoms not serious enough, with the effect of location being not a deterring reason [39].

### Hygiene practices and latrine use

In addition, the current study found out that the study participants were aware that it was beneficial to own and utilize pit-latrines. Nevertheless, majority of the respondents reported not to own and utilize pit latrines. Hence villagers were practicing open defecation due to factors like high building cost, availability of large pieces of land and cultural beliefs, whereby it is a taboo for a father in-law to share a latrine with a daughter in-law. Acka and colleagues reported a similar practice of poor hygiene, where villagers tended to defecate where convenient still rarely using latrines where available [31]. This practice allows helminthes eggs from the feces of infected persons to contaminate the environment including water sources hence the community members. In this regard, it would be interesting to try a community led total sanitation approach in the study area. (CLTS) [40]. CLTS is an integrated approach to achieve and sustain an open defecation-free status of community members through a participatory approach. This method of CLTS can facilitate participation of community members in the study area to improve sanitation, hygiene practices, waste disposal and protection of drinking water sources [41].

## Study limitation

The major potential weakness of this study may be the fact that the results are not generalizable to other parts of the country as this was a qualitative study that was used to explore and assess KPP. However, the current study could be used to develop theory through tentative hypotheses.

## Conclusions and recommendations

In conclusion, our results show that to large extent participants had knowledge and information on worm infection. However, some beliefs on the etiology, pathology and mode of transmission were mentioned that could hinder control efforts in the ongoing programmes. Accordingly, other approaches towards enhancing health outcomes in the community could be used like implementing community MDAs for improved control. In addition, health education to the community members on the importance of owning and utilizing pit latrine to reduce open defecation which is highly practiced in the study area would really be of much help. While putting into consideration the underlying cultural beliefs and practices that hinder them from proper human waste disposal. Results of this study can be used to inform the design of appropriate health education messages, for which one needs to first understand what the gaps are in the knowledge that already exists. In addition, there were misconceptions and misperceptions that needed to be addressed about the ineffectiveness of the treatment given by the NSBDP for improved control by education the community members. Our results, further suggest that improved access to clean water and sanitation are necessary for sustainable control of major helminthiasis in the study area and other endemic parts of the country.

## Supporting information

S1 Appendix Strobe check list. (DOC)

S2 Appendix Focus group discussion guide. (DOCX)

S3 Appendix Social demographic profile. (DOCX)

S4 Appendix Consent form. (DOCX)
