# Supplementary material for: Knowledge, practices and perceptions of geo-helminthes infection among parents of pre-school age children of coastal region, Kenya
Source: PLoS Negl Trop Dis. 2017 Mar 30;11(3):e0005514. doi: 10.1371/journal.pntd.0005514 (PMC5388494; doi:10.1371/journal.pntd.0005514)
Supplement: S2 Appendix — (DOCX) [file pntd.0005514.s002.docx]

**Supporting Information**

**Text S1 Focus group discussion guide**

**Focus Group Discussion for Parents of ECD Children on knowledge on Intestinal Parasites and the National School-based deworming programme knowledge and awareness Survey**

| Instructions:   - This form should be used for FGDs with parents of ECD Children. - If the participants refuse to answer a question, circle the number of the question and do not mark any answers for that question. - Each FGD MUST have 8-12 participants - After obtaining written informed consent, read the following instructions to the participants: |
| --- |
| **“I am going to ask you questions about the National School -Based Deworming Programme so as to collect information about your knowledge, attitudes and practices on Bilharzia and soil transmitted helminthes, the Control Programme**, **your opinion of the Programme, your community members willingness for their children to participate in the programme and their preferences for being reached during the Programme. Please answer the questions as honestly as you can remember. Your information which will be tape recorded will be kept private and this form will not have your name anywhere. If you have any questions or do not understand what I am asking you at any time, please ask for clarification. Some questions may prove embarrassing to you.**  **Please remember that you do not have to answer any questions that you do not want to answer and you may discontinue the discussion at any time. Do you have any questions before we begin?”** |

**Ice breaker**

Let us now begin. Let’s find out some more about who we are. You will tell us your name and introduce yourself very briefly, for example where you live and anything else you would like to mention about yourself.

**[→Start recorder after this has been done]**

**Focus Group Questions**

**Intestinal Parasites knowledge and awareness Survey**

1.What are the common diseases in your village? If intestinal worms not mentioned ask- Are intestinal worm infections among the common diseases in your region? Probe for:

- Types of worms (round worms, tapeworms, bilharzia)
- Local name.

2. Where did you first learn about intestinal worms? Probe for:

- Newspapers and magazines
- Mass media: Radio; TV; Billboards
- Brochures, posters and other printed materials
- Professionals: Health workers; Teachers
- Family, friends, neighbours and colleagues
- Leaders: At *barazas*; Religious leaders
- Other (please specify):

3. What are the signs and symptoms of intestinal worms? Probe for:

- Symptoms associated with different types of intestinal worms

4. What would you say about diagnosis of intestinal worms? Probe for:

- Diagnostic methods
- Acceptability
- Accessibility
- Affordability- cost
- Effectiveness

5. How does a person get intestinal worms? Probe for:

- Life cycle
- Perceived Cause
- How it spreads

6. Who are most at risk of getting intestinal worms? Probe for;

- Age group
- Gender
- Risky behaviors
- Occupation hazards
- What are the reasons which make you think so?

7. How can intestinal worms be avoided? Probe for:

- Preventive and control measures
- Current interventions by community, government, others.
- What is perceived to have worked in the past and the reasons for such perceptions
- What is perceived to have failed and the reasons for such perceptions
- Acceptability
- Accessibility / Reach (define)
- Affordability

8. Are intestinal worm infections curable? Probe for:

- Available drugs?(ALB, MBZ, PZQ)
- Cost of the drugs, Exact cost figures (perceived or real)
- How perceived (or real) costs may influence care seeking
- Herbal remedies
- Home rest without medicine
- Praying
- Any other method.
- Effectiveness of methods stated reasons for this perception

9. In your opinion, how serious a disease are intestinal parasites? Probe for:

- How serious a problem do you think intestinal parasites are in this region?
- What prompts you to think this way/ please elaborate your answer?

10. Earlier at the beginning of our discussion you listed some of your sources of information about intestinal parasites. Do you feel well informed about intestinal parasites? Probe for

- What are your other sources of information?
- How adequate do you think these sources are?
- If you could get more information about intestinal parasites, what additional information would you wish to get?
- Preferred source of information.
- Most accessible source. What makes you say this source is more accessible?

**Attitude on Intestinal Parasites**

11. Do you think you or members of your family can get intestinal parasites? Probe for:

- What makes you feel you are either at risk, or not at risk of getting intestinal worms?

12. What would be your reaction if you found out that you or a member of your family has intestinal parasites? Probe for:

- reasons for different reactions (fear, surprise, embarrassment etc)

13. Who would you talk to about your illness if you had intestinal parasites? Probe for:

- - - - Doctor or other medical worker
- Spouse
  - - - Parent
- Child(ren)
- Other family member
- Close friend
- No one
- Other:

Probe for:

- - - What would prompt you to talk about your illness?
    - What would make you choose a specific person to talk to about your illness?

14. Do you know people who have/had intestinal parasites? Probe for:

- What makes you say they have intestinal worms

15. In your community, how is a person who has intestinal parasites usually regarded/treated?

**Health- seeking practices**

16. Would you go or take family members to the health facility if you suspect you have intestinal parasites? Probe for:

- Place of treatment
- People to seek treatment at the places you have mentioned?
- Are certain care options more likely to be used than others?
- Under what circumstances does this happen?
- Self-medication

17. If you or member of your family had symptoms of intestinal parasites, at what point would you go to the health facility? Probe for:

- Low
- Moderate
- Severe

18. If you would not go to the health facility, what is the reason? Probe for:

- Not sure where to go
- Cost
- Difficulties with transportation/distance to clinic
- Do not trust medical workers
- Do not like attitude of medical workers
- Cannot leave work (overlapping work hours with medical facility working hours)
- Do not want to find out that something is really wrong
- Pursue other self-treatment options (herbs, etc.)
- Go to pharmacy
- Go to traditional healer

19. What worries you the most when you think about intestinal parasites?

**Awareness and attitude of School-based deworming**

20. Have you heard about school-based deworming for intestinal parasites in your region?

Probe about

- Has it been conducted
- When it was conducted
- Where it was conducted
- Length of deworming activity
- Mode of drug distribution and
- Interaction with drug administrator/teacher/CHEW.

21. How do you (and people in your village) feel about the School-based deworming activity? Probe for

- Importance of the school-based deworming activity
- The length of the school-based deworming activity?

22. How did people in your village learn about the school-based deworming activity? Probe for

- Sources of information
- Sufficiency in informing the communities about the school-based deworming activity
- frequency of informing the communities about the school-based deworming activity
- effectiveness of the source of information

23. Did your ECD child/children take the drugs during the school-based deworming activity?

Probe for:

- How did you as a parent support the deworming activity (providing safe water, feeding the children before treatment, escorting them to and from ECD centres on deworming day)
- Preferred method to use in administering treatment to ECD children next time offered

24. Did your ECD child/children experience any problems after taking the drugs for worms?

Probe for:

- Kind of problems
- How resolved
- Possibility of taking drugs next time offered

25. What is your opinion regarding the school-based deworming activity for intestinal worms

Probe for

Importance/need

Effectiveness

**THANK YOU VERY MUCH FOR YOUR COOPERATION**
