# Supplementary material for: Knowledge, practices and perceptions of geo-helminthes infection among parents of pre-school age children of coastal region, Kenya
Source: PLoS Negl Trop Dis. 2017 Mar 30;11(3):e0005514. doi: 10.1371/journal.pntd.0005514 (PMC5388494; doi:10.1371/journal.pntd.0005514)
Supplement: S3 Appendix — (DOCX) [file pntd.0005514.s003.docx]

Text S3 Social demographic profile

**Socio-Demographic Characteristics**

| FGD category: | | | Moderator: | | |
| --- | --- | --- | --- | --- | --- |
| FGD location: | | | Note taker: | | |
| Date of FGD: | | | Time start: | | |
| Location of FGD: | | | Time stop: | | |
| No. Of participants at stop | | | Observer (s) | | |
| **Participant** | **Age** | **Sex** | **Ed (yrs)** | **Religion** | **Occupation** |
| 1 |  |  |  |  |  |
| 2 |  |  |  |  |  |
| 3 |  |  |  |  |  |
| 4 |  |  |  |  |  |
| 5 |  |  |  |  |  |
| 6 |  |  |  |  |  |
| 7 |  |  |  |  |  |
| 8 |  |  |  |  |  |
| 9 |  |  |  |  |  |
| 10 |  |  |  |  |  |
| 11. |  |  |  |  |  |
| 12. |  |  |  |  |  |
