# Supplementary material for: Knowledge, practices and perceptions of geo-helminthes infection among parents of pre-school age children of coastal region, Kenya
Source: PLoS Negl Trop Dis. 2017 Mar 30;11(3):e0005514. doi: 10.1371/journal.pntd.0005514 (PMC5388494; doi:10.1371/journal.pntd.0005514)
Supplement: S4 Appendix — (DOCX) [file pntd.0005514.s004.docx]

**Text S3 Consent form.**

**Title of Study:**

Evaluating Different Drug Delivery Approaches for Treatment of Soil-transmitted Helminthiasis

and Schistosomiasis Infections in the National School-based Deworming Programme among

Children Attending Early Childhood Development (ECD) Centers in Coast Province, Kenya.

**Sponsor:** NATIONAL COMMISSION FOR SCIENCE, TECHNOLOGY AND INNOVATION

**Principal Investigator:**

Doris Wairimu Njomo, PhD Public Health

ESACIPAC, KEMRI

**Introduction**

You are asked to participate in a social research study on intestinal worms. Intestinal worms are a group of parasitic worms causing human infection through contact with parasite eggs or larvae that thrive in the warm and moist soil or water. As adult worms, they live for years in the human body. Of particular worldwide importance are the roundworms, whipworms, bilharzia and hookworms. They are considered together because it is common for a single individual, especially a child living in a less developed country, to be chronically infected with all four worms. Such children have malnutrition, growth stunting, intellectual retardation, and cognitive and educational deficits. The most common way to find out if someone has these worms is to check for the eggs of the parasite in the stool or urine. Children are most at risk of these worm infections and they suffer most when/if infected.

The government of Kenya has decided to control these intestinal worms by giving treatment (through the National School-based Deworming Programme) to all children (2-14 years) living in areas where the infections levels are high. The purpose of this consent form is to give you information that might help you to decide whether to participate in the study or not. You are allowed to ask questions related to the study and implications on your part.

**Purpose of study**

The government of Kenya is conducting school-based deworming for intestinal worms using primary schools as the treatment centers and primary school teachers as the drug administrators/distributors. All parents and teachers of children attending ECD centers are advised to move their children to a nearby primary school to get the drugs during the treatment day.Scientists still do not know if this is the best way of reaching the ECD children. If some infected children do not get treated at primary schools during the Programme, they serve as source of reinfection and thus the expected reduction of force of transmission will not be achieved. We are therefore interested to find out the best method of making sure that all or as many as possible ECD children who are at risk of getting intestinal worms get the treatment, which is available. As such some ECD Center children were treated at their Centers by the Community Health Extension Workers while others were treated by the primary school teachers at the nearest primary school. This will help the government through the National School-Based Deworming Program achieve its objective of treating all children in the age of 2-14 years for control the intestinal parasites.

**Procedures to be followed**

As a community member/ teacher/ leader/ CHEW you will be asked to give information on your knowledge about drug administration, your participation in the School-based Deworming Programme, your willingness to have your child continue taking the drugs and your suggestions for improved deworming process. They will take pictures of activities in the community and take notes, which will help us to explain our findings. They will talk to women and men in this community, either in groups or individually. Some of the conversations will be tape recorded, so that we do not miss out some of the important things that will be said.

**Benefits**

The aim of this study is to identify, design and test an alternative strategy that can be used for increased treatment coverage for intestinal parasites among ECD aged children. The information you give will help come up with improvement measures aimed at improving and raising the levels of treatment coverage. In the long run you and the members of your community will not be at high risk of intestinal worms.

**Risks**

There is no risk of participation in this study. You will not be expected to give your names to the person collecting data from you.

**Assurance of confidentiality**

The information you give and other records about you will remain confidential and will not appear when we present this study or publish its results. You will receive a copy of the consent form.

**Storage of data**

The data will be stored in secure cabinets and computers with password/s and will only be accessible to the investigators.

**Right to refuse or withdraw**

It is important that you understand the following general principles that will apply to all participants in the study:

1. Participation is entirely voluntary.

2. You may withdraw from this study at any time without penalty or loss of benefits.

Please feel free to ask any questions that you may have. Do you agree to participate?

I acknowledge that this consent form has been fully explained to me in a language that I understand and had the opportunity to ask questions which have been answered to my satisfaction. I agree voluntarily to participate in this study and understand that I have the right to withdraw at any time without penalty.

Participant's name: _____________________________________________

Participant's signature or thumb print: ________________________

Date: ____________

Study No.: SSC 2547

Name of witness: _____________________________________________

Signature of witness: ____________________________ Date: ___________________

Investigator's signature: __________________________­­­­ Date: ___________________

**Contact:** If you have questions in future, please contact **The Secretary, KEMRI/National Ethical Review Committee, P. O. Box 54840-00200, Nairobi, Telephone 020-2722541 Ext 3307** or Dr. Doris W. Njomo, Kenya Medical Research Institute, Eastern and Southern Africa Centre of International Parasite Control **(ESACIPAC)**; Telephone 0722373650.
